# MicroRNA-155-3p promotes hepatocellular carcinoma formation by suppressing FBXW7 expression

**DOI:** 10.1186/s13046-016-0371-6

**Published:** 2016-06-16

**Authors:** Bo Tang, Biao Lei, Guangying Qi, Xingsi Liang, Fang Tang, Shengguang Yuan, Zhenran Wang, Shuiping Yu, Songqing He

**Affiliations:** Department of Hepatobiliary Surgery, Guilin Medical University, Affiliated Hospital, Guilin, 541001 Guangxi People’s Republic of China; Laboratory of Liver Injury and Repair Molecular Medicine, Guilin Medical University, Guilin, 541001 Guangxi People’s Republic of China; Department of Pathology and Physiopathology, Guilin Medical University, Guilin, 541004 Guangxi People’s Republic of China

**Keywords:** FBXW7, Hepatocellular carcinoma, miR-155-3p

## Abstract

**Background:**

MicroRNAs (miRNAs) are small non-coding RNAs frequently dysregulated in human malignant tumors. In the present study, we analyzed the role miR-155-3p plays in Hepatocellular carcinoma (HCC), which has been reported participation in some other types of cancer.

**Methods:**

qRT-PCR was used to measure the levels of miR-155-3p in HCC specimens and HCC cell lines. Overexpression of miR-155-3p and miR-155-3p inhibitor were transfected into HCC cell lines to investigate its role in HCC. Colony formation assay and 3-(4, 5-dimethylthiazol-2-yl)-2,5-diphenyltetrazolium (MTT) assays were used to analyses cell proliferation in vitro. In vivo tumor formation assays were performed in BALB/c nude mice. Luciferase reporter assay was carried out to measure the translation of F-Box and WD repeat romain containing 7 (FBXW7).

**Results:**

We found that miR-155-3p was remarkably upregulated both in HCC tissue and cell lines. Overexpression of miR-155-3p enhanced HCC cell proliferation in vitro and tumorigenesis in vivo. In addition, overexpression of miR-155-3p is correlated with decreased levels FBXW7 mainly through inhibiting the expression of FBXW7.

**Conclusions:**

Our studies suggest that miR-155-3p plays an important role in the pathogenesis of HCC and implicates its potential applications in the treatment of HCC cancer.

## Background

Hepatocellular carcinoma (HCC) is one of most common malignant tumor worldwide and ranks third of mortality rate, with about 500,000 new cases annually [1]. The major risk factors for HCC includes the presence of cirrhosis, Hepatitis B virus/hepatitis C virus (HBV/HCV) infection, and other factors, such as nonalcoholic steatohepatitis, are common in certain areas of the world [2]. HCC is the second most common mortality factor of cancer and is characterized with late diagnosis, poor prognosis, as well as metastatic tendency and insensitivity to chemotherapy and radiotherapy [3]. Previous studies found that the occurrence of liver cancer is a slow process with gradual changes that develop mainly as a result of chronic hepatitis and hepatic fibrosis. Those pathological processes suggest gene and protein expression alterations [4].

MicroRNAs (MIRs) are endogenous non-coding RNAs which contain 18 to 25 nucleotides and play important roles in regulating gene expression [5]. The mature forms of MIRs silence the gene expression is binding to the 3’-untranslated region (UTR) of target mRNAs and initiate the translational repression or cleavage of cognate mRNAs [6, 7]. miRNAs have frequently been implicated in carcinogenesis [8–11]. In the setting of HCC, miR-222 [12], miR-21 [13], miR-106b [14] and miR-331-3p [15] have been reported to be tumor oncogene.

Among the known oncomirs, miR-155 stands out as an important entity. It is one of the most commonly up-regulated miRNAs in tumors [16]. Furthermore, miR-155 has been reported as an oncomir in various human cancers, including colorectal [17], glioma [18], esophageal [19], liver [20], oral squamous [21] and lymphatic system [22]. miR-155-5p and miR-155-3p, two different miRNA strands, produced from the miR-155 host gene produces,. The miR-155-5p has been considered as the only functional miR-155 form [23]. Previous studies found that miR-155-3p is also strongly upregulated in T cells. Functional manipulation of miR-155-3p expression revealed its important role in regulating Th17 development. The search for miRNA-155-3p target genes highlighted transcripts of two heat shock protein 40 genes, Dnaja2 and Dnajb1 [24]. Such exploration is likely to provide important information regarding the miR-155-3p signature and their target genes at a very early stage of liver tumorigenesis and their relationship to the miRNA signature of primary human HCC that can be used in the diagnosis and prognosis of liver cancer.

F-box and WD repeat domain containing 7 (FBXW7) protein encodes a substrate adaptor for an E3 SCF ubiquitin ligase complex and negatively regulates the abundance of different oncoproteins [25]. Many observations indicate that FBXW7 lies at the nexus of many pathways include controlling cell growth, cell differentiation, and tumor genesis. FBXW7 gene is further supported as a human tumor suppressor by the discovery of FBXW7 gene mutations in cancers from a wide spectrum of human tissues [26]. A recent study showed that colorectal cancer patients with low FBXW7 levels had poorer prognoses [27].

In this study, we aimed to investigate whether miR-155-3p is an oncomir in human HCC and identify the direct target correlated with the malignant phenotype of HCC. We demonstrated that miR-155-3p upregulating was a frequent event in HCC tissues and could be a potential targets for HCC patients. Furthermore, our findings also showed that ectopic expression of miR-155-3p could accelerate clone formation and proliferation ability of HCC cells. In addition, we further identified FBXW7 as a functional target of miR-155-3p and demonstrated FBXW7 involve in the effects of increased miR-155-3p on promoting clone formation and proliferation. Our data suggest a fundamental role for miR-155-3p in clone formation and HCC cells proliferation, and implicate the potential application of miR-155-3p in prognosis prediction therapy of liver cancer.

## Methods

### Patients and specimens

Hepatocellular carcinoma tumor tissues and normal liver tissues (para-cancerous tissues) were randomly collected from HCC patients who underwent curative resection with informed consent between 2012 and 2014 at the Department of Hepatobiliary Surgery, Affiliated Hospital of Guilin Medical University. All tissues were collected immediately upon resection of the tumors in the operation theater, transported in liquid nitrogen, and then stored at -80 °C.We set the samples on the slide glass and microscopically recognized the malignantly transformed epithelial lesion by H&E staining, then cored out the epithelial lesion. Study protocols were approved by the Hospital Ethics Committee of Guilin Medical University, and written informed consent was obtained from patients based on the Declaration of Helsinki.

### qRT-PCR

Total RNA was extracted using TRIzol Reagent (Invitrogen, Carlsbad, CA, USA). The miR-155-3p and U6 levels were quantified using qRT-PCR with the TaqMan® Micro-RNA Reverse Transcription Kit (Applied Biosystems, Foster City, CA, USA) and TaqMan® MicroRNA Assays (Applied Biosystems) according to the manufacturer’s instructions. We assessed the RNA expression according to relative quantification using the 2^-ΔΔCt^ method to determine the fold change in the expression. The primers used for the expression analysis were as follows: GAPDH-forward, 5′-C TCATGACCACAGTCCATGC-3′: GAPDH-reverse, 5′- TTACTCCTTGGAGGCCATGT-3′: U6-forward, 5′- CTCGCTTCGGCAGCACA -3′: U6 - reverse, 5′- AACGCTTCACGAATTTGCGT 3′. FBXW7 - forward, 5′- GGG AGCACTTTGCTGAAATC-3′: FBXW7 - reverse, 5′- CAGCAGCCACTTCTTGAAAC -3′.

### miR-155-3p measurement

miR-155-3p levels were determined by two-step real time-PCR. Reverse transcription reaction was performed with specific microRNA primers. Real time PCR amplification was carried out with a Rotorgene 3000 machine (Corbett). Relative microRNA concentrations are given as the ratios between the amount of the target gene and the endogenous control U6.

### Cell lines

The human HCC cell lines THLE-3, HepG2, Hep3B and SUN475 were obtained from RIKEN BioResource Center (Tsukuba, Japan) and maintained in Dulbecco’s Modified Eagle’s Medium (DMEM) with 10 % FBS, 2 mM L-glutamine and 100 U/ml of penicillin and streptomycin in a 6-cm dish. BEL-7405, BEL-7404 and BEL-7402 were obtained from the ATCC (Manassas, VA, USA) and maintained in Mc-Coy’s 5a Medium with 10 % FBS, 2 mM L-glutamine and 100 U/ml of penicillin and streptomycin and in Roswell Park Memorial Institute medium 1640 with 10 % FBS, 2 mM L-glutamine and 100 U/ml of penicillin and streptomycin.

### Overexpression of miR-155-3p

Precursor- miR-155-3p was transfected into BEL-7405 using the BLOCK-iT™ Lentiviral miR RNAi Expression System (Invitrogen, Carlsbad, CA, USA) following the manufacturer’s protocol, as previously described. After transfection, we performed blasticidin selection at a concentration of 2.5 μg/ml for 10 days.

### MiR inhibitor

A total of 200nM of microRNA Hairpin Inhibitor and its negative control (Thermo Scientific Dharmacon, Lafayette, CO, USA) were employed to transiently inhibit miR-155-3p and transfected 48 h prior to seeding with Oligofectamine (Invitrogen).

### Colony formation assay

A total of 1.0 × 10^5^ cells were seeded in a layer of 0.4 % noble agar/DMEM/1 % FBS/0.5 μg/ml of puromycin or 0.4 % noble agar/DMEM/5 % FBS over a layer of 0.5 % bactoagar/DMEM/1 % or 5 % FBS in a 6 cm dish. The colonies were stained using 3-[4,5-dimethylthiazol-2-yl]-2,5-diphenyltetrazolium bromide solution (Sigma-Aldrich, St. Louis, MO, USA) and counted.

### Cell proliferation assay

Cells were seeded in 96-well plates in triplicate at densities of 1 × 10^3^ per well. Cell proliferation was monitored at desired time points using 3-(4, 5-dimethylthiazol-2-yl)-2, 5-diphenyltetrazolium bromide (MTT) (Promega). In brief, the MTT assay was performed by adding 10 μl MTT (10 mg/ml) for 4 h. Light absorbance of the solution was measured at 570 nm on a microplate reader.

### Analysis of the tumor-forming potential in vivo

All experiments were conducted in accordance with guidelines authorized by the Animal Research Committee of Guilin Medical University. Six-week-old BALB/c nude mice were injected subcutaneously into their flanks with 2 × 10^7^ BEL-7405 mock or BEL-7405 miR-155-3p cells bilaterally in 200 μl of normal culture medium. All mice were sacrificed on day 28, and the tumor weight was measured.

### Immunoblotting

SDS-PAGE and immunoblotting were carried out as described elsewhere [28]. Briefly, filters were incubated with rabbit polyclonal antibodies against FBXW7, mouse monoclonal antibodies against β-actin (1:2,000 dilution, Abcam, Cambridge, UK).

### Luciferase reporter assay

To investigate the translation of FBXW7, luciferase reporter assay was carried out as described [29]. The wild-type or mutant FBXW7 3’-UTR sequence was inserted downstream of the firefly luciferase reporter gene, which was controlled by the SV40 enhancer for expression in mammalian cells, whereas no oligonucleotides were inserted in the control vector (Genecopoeia, Rockville, MD, USA). Renilla luciferase was used as a tracking indicator for successful transfection. In order to investigate the transcription of FBXW7, luciferase reporter constructs for the promoters of these molecules and a positive control of glyceraldehyde-3-phosphate dehydrogenase (GAPDH) were obtained (SwitchGear Genomics, Menlo Park, CA, USA). The luciferase activity was measured using Light- Switch Assay Reagent (SwitchGear Genomics) according to the manufacturer’s instructions. Briefly, 1.0 to 1.5 × 10^4^ cells were seeded in white 96–well plates on day 1 and transfected with reporter constructs on day 2 using FuGENE HD (Promega). The luciferase activity was measured using assay reagent 48 h after transfection.

### Overexpression of FBXW7

FBXW7 lentiviruse (Sigma-Aldrich) was transfected into BEL-7405-miR-155-3p cells in 48-well plates according to the manufacturer’s instructions. The multiplicity of infection (MOI, number of transducing lentiviral particles per cell) was 5. We performed puromycin selection at a concentration of o.5 μg/ml for 10 days.

### RNA interference for FBXW7

One siRNA lentiviruse against FBXW7 (Sigma-Aldrich) and non-targeting siRNA (Sigma-Aldrich) were transfected into HepG2-Anti-miR-155-3p cells in 48-well plates according to the manufacturer’s instructions. The multiplicity of infection (MOI, number of transducing lentiviral particles per cell) was 5. We performed puromycin selection at a concentration of 0.5 μg/ml for 10 days.

### Immunohistochemistry (IHC)

Formalin-fixed, paraffin-embedded tissues were used to detect the FBXW7 expression. The sections were incubated with anti-FBXW7 rabbit polyclonal antibodies (Abcam, Cambridge, UK) at 1:300 dilution. A semi-quantitative scoring system was used to evaluate the intensity of staining: low (proportion: 0 to 50 %, intensity: no staining to weak) and high (proportion: more than 50 %, intensity: intermediate to strong).

### Microarray

RNeasy Lipid Tissue Mini kit (Qiagen) was used to isolate total RNA from the different cells according to the manufacturer’s protocol and RNA were stored in liquid N2 at -80 °C until further processing. The quantity and quality of RNA were assessed using a NanoDrop ND-1000 spectrophotometer and an Agilent Bioanalyzer and samples with an RNA Integrity number (RIN) > 7 were only used for further analysis. For microarray hybridizations, 100 ng of total RNA was amplified and labeled using the MessageAmp Premier Kit (Ambion). Equal amounts of labeled cRNA were hybridized to the Affymetrix Genome 2.0 microarray (Affymetrix) according to the manufacturer’s protocol. Partek Genomics Suite 6.4 (Partek Inc., St. Louis, MO) was used to perform data analysis. Robust multi-chip analysis (RMA) normalization was done on the entire data set. Multi-way ANOVA and fold change were performed to select target genes that were differentially expressed between the different comparisons. Top differentially expressed genes were selected with p value cut-off of 0.05 based on ANOVA test and fold change cut-off of ≥ 2. Gene Ontology Enrichment analysis on the gene lists were performed with chi-square test and limited to functional groups with more than two genes. Hierarchical Clustering was performed on differentially expressed genes based on Average Linkage with Pearson’s Dissimilarity. Additionally, the gene lists were analyzed using the GeneGo software for obtaining pathway maps, biological networks and diseases relevant to the list.

### Statistical analysis

The data are presented as the mean ± SEM. The unpaired two-tailed Student’s t-test, Mann-Whitney’s Utest and Chi-square test were used for comparisons, with a p value of < 0.05 considered to be significant (*).

## Results

### Elevated expression of miR-155-3p in human hepatocellular carcinoma tissues and cell lines

**Fig. 1:**
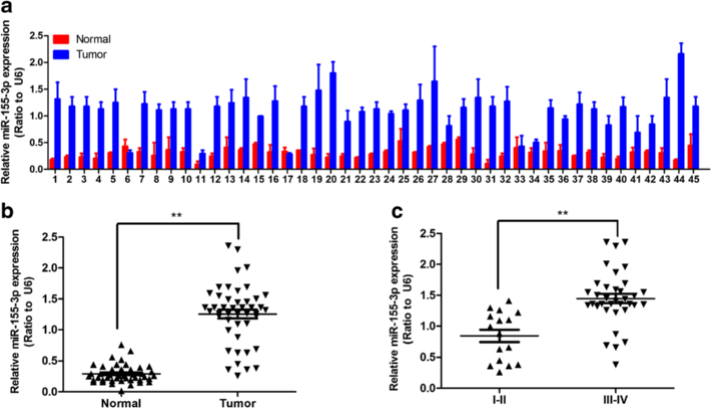
The expression of miR-155-3p is elevated in Hepatocellular carcinoma tissues. **a** miR-155-3p expression in Hepatocellular carcinoma in comparison to normal tissues was measured by qRT-PCR. **b** miR-155-3p expression levels are significantly elevated in Hepatocellular carcinoma in comparison to normal tissues. **c** miR-155-3p expression levels are significantly increased in patients of stage III-IV in comparison to the stage I-II. **P < 0.01, unpaired two-tailed Student’s t-test

**Fig. 2:**
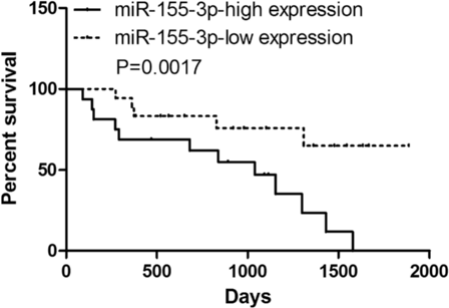
Elevated expression of miR-155-3p is associated with poor patient survival of Hepatocellular carcinoma

In order to investigate whether the miR-155-3p expression is correlated with the HCC tumorigenesis, we examined miR-155-3p expression levels in a panel of 45 human HCC tissues (Fig. 1a). We found that the level of miR-155-3p was elevated in most of the malignant HCC tissues by 1.5 to 6 fold as expected (Fig. 1b). To investigate the clinical value of elevated miR-155-3p expression in HCC, we assessed the association between miR-155-3p expression levels and clinical significance in HCC tissues samples. MiR-155-3p expression levels are significantly elevated in patients of stage III-IV in compared with the stage I-II (Fig. 1c). To determine the prognostic impact of miR-155-3p expression in HCC, we categorized HCC patients into two groups based on miR-155-3p expression levels. Patients with tumors displaying high miR-155-3p expression levels had significantly shorter percent survival compared with those with low miR-155-3p (Fig. 2).

### The overexpression of miR-155-3p in BEL-7405 cells enhances tumorigenesis in vitro and in vivo

**Fig. 3:**
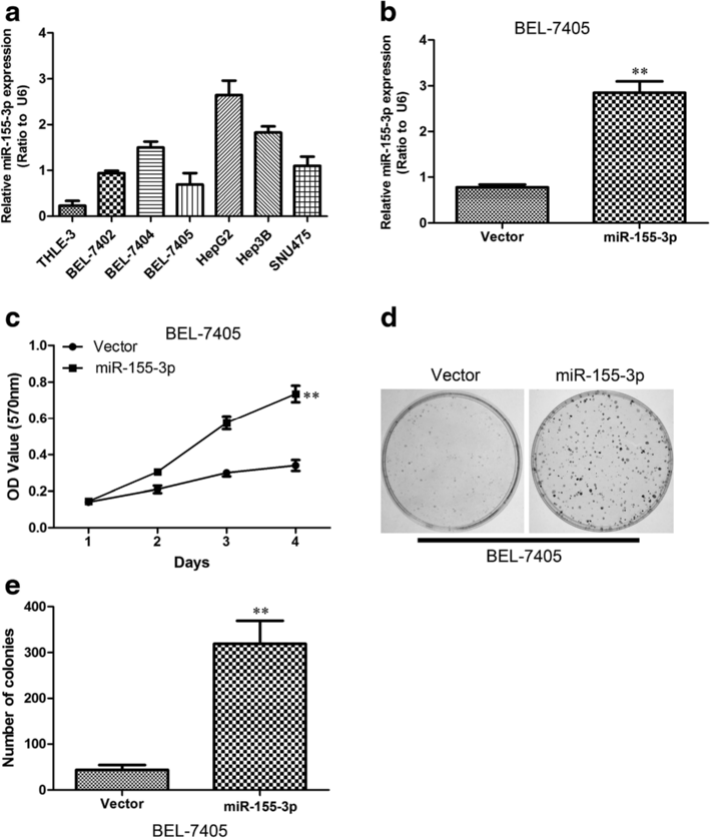
The overexpression of miR-155-3p enhances tumorigenesis in vitro. **a** Expression profile of miR-155-3p in Hepatocellular carcinoma cell lines. miR-155-3p RNA levels relative to normal liver cell line THLE-3 were determined by qRT-PCR. Gene expression was normalized to U6. Data are presented as means ± Standard deviation. **b** Establishment of BEL-7405-expressing miR-155-3p cells. The results were analyzed by qRT-PCR. **c** Proliferation of BEL-7405-miR-155-3p cells is significantly accelerated compared to normal BEL-7405 control cells measured by MTT assay. **d** Representative stained colonies are displayed. **e** The number of colonies was counted. **P < 0.01, unpaired two-tailed Student’s t-test

Then we examined miR-155-3p expression levels in a panel of 7 widely used human HCC cell lines in comparison to levels in non-malignant cell lines THLE-3(Fig. 3a). Correspondingly, miR-155-3p expression levels are consistently elevated in HCC cell lines. Since miR-155-3p is overexpressed in human HCC tissues and cancer cell lines, we considered whether miR-155-3p functions as an oncomir. We established BEL-7405 cells overexpressing miR-155-3p by introducing precursor-miR-155-3p using lentivirus vectors because the miR-155-3p expression level in BEL-7405 cell was the lowest among the several HCC cell lines analyzed and lentivirus vectors can be efficiently transfected into this cell line. The presence of a mature-miR-155-3p expression was confirmed by using qRT-PCR (Fig. 3b).

**Fig. 4:**
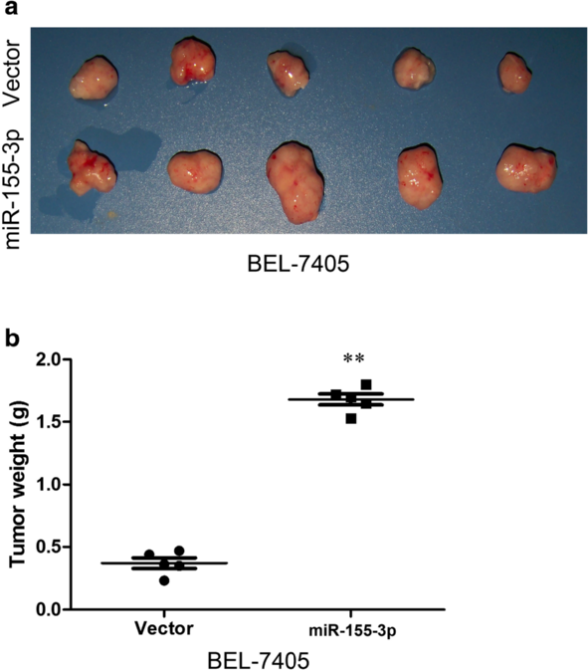
The overexpression of miR-155-3p enhances tumorigenesis in vivo. **a** Subcutaneous tumors in the five nude mice are displayed. **b** The weights of tumors are shown. **P < 0.01, unpaired two-tailed Student’s t-test

To corroborate this assumption, we tested whether overexpression of miR-155-3P in BEL-7405 cells promotes cell growth and colony formation ability by using MTT and colony formation assay. Overexpression of miR-155-3p strongly stimulates cell proliferation compared to control cells indicating that miR-155-3p is potentially oncogenic (Fig. 3c). In addition, it significantly promoted colony formation (Fig. 3d and e). The miR-155-3p mediated tumorigenic effects were confirmed in an in vivo model. A significant increase in tumor weight was observed in the BEL-7405 cells with miR-155-3p overexpression compared with that noted in the controls in the nude mice subcutaneous tumor model (Fig. 4a). A dramatic increase in tumor growth was observed in BEL-7405-miR-155-3p compared with the BEL-7405-vector (Fig. 4b). These findings demonstrate that miR-155-3p can promote HCC cell proliferation in vitro and in vivo.

### Inhibition of miR-155-3p in HepG2 cells reduces tumorigenesis in vitro and in vivo

**Fig. 5:**
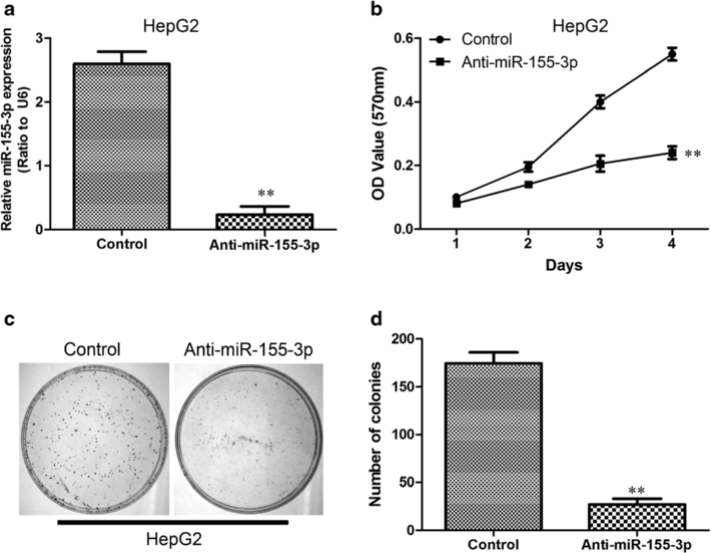
Downregulation of miR-155-3p reduce tumorigenesis in vitro and in vivo. **a** Establishment of downregulation of miR-155-3p in HepG2 cells. The results were analyzed by qRT-PCR. **b** Proliferation of HepG2-miR-155-3p cells is significantly accelerated compared to normal HepG2 control cells measured by MTT assay. **c** Representative stained colonies are displayed. **d** The number of colonies was counted. **P < 0.01, unpaired two-tailed Student’s t-test

Since the miR-155-3p expression level of HepG2 was highest among the several HCC cell lines analyzed, we suppress miR-155-3p expression in HepG2 cells by introducing Anti- miR-155-3p using lentivirus vectors and lentivirus vectors can be efficiently transfected into this cell line. The presence of a mature-miR-155-3p expression was confirmed by qRT-PCR (Fig. 5a).

**Fig. 6:**
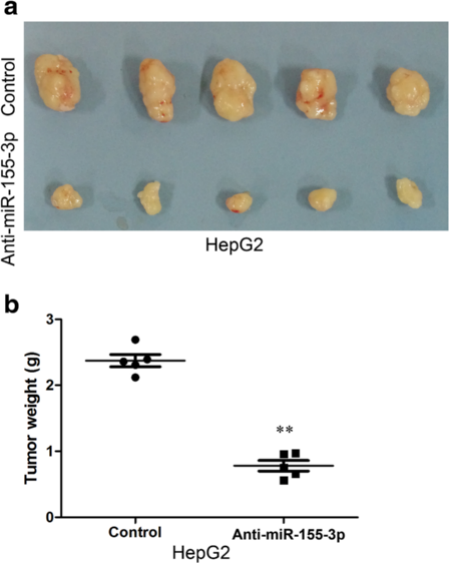
Downregulation of miR-155-3p reduce tumorigenesis in vivo. **a** Subcutaneous tumors in the five nude mice are displayed. **b** The weights of tumors are shown in the right graph. **P < 0.01, unpaired two-tailed Student’s t-test

Then we tested whether downregulation of miR-155-3p in HepG2 cells reduces cell growth and colony formation ability. Downregulation of miR-155-3p can strongly inhibit cell proliferation (Fig. 5b) and significantly restrains colony formation compared to control cells (Fig. 5c and d). The miR-155-3p mediated tumorigenic effects were confirmed in an in vivo model (Fig. 6a). A significant reduces in tumor weight was observed in the miR-155-3p downregulated HepG2 cells compared with that noted in the controls in the nude mice subcutaneous tumor model (Fig. 6b). These findings demonstrate that miR-155-3p induces a more aggressive phenotype of HCC.

### MiR-155-3p reduces the protein levels of FBXW7 by inhibiting translation

**Fig. 7:**
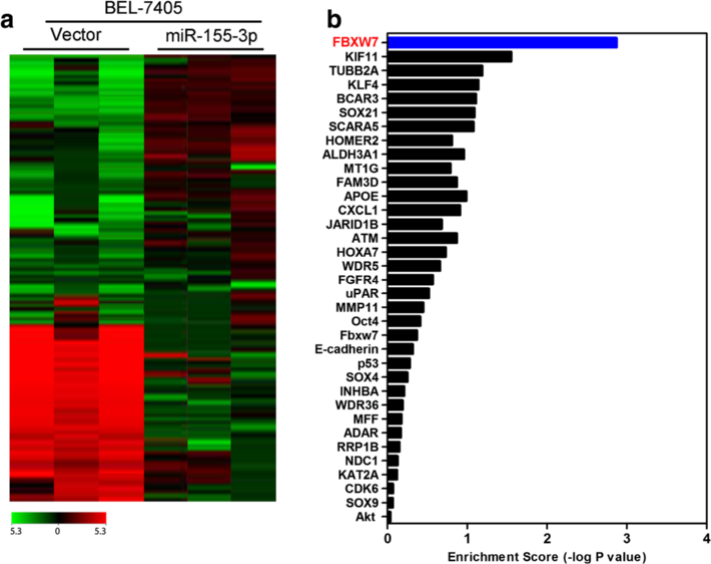
miR-155-3p regulates the expression of FBXW7. **a** Supervised hierarchical clustering of the genes differentially expressed after miR-155-3p overexpression in BEL-7405 cells. **b** Gene set enrichment analysis was carried out using ConceptGen

To better characterize the mechanisms by which miR-155-3p engaged in HCC development and progression, microarray assay was performed in cell line BEL-7405-miR-155-3p and its control cells with the empty plasmid. Microarray results indicated that a list of genes expression significantly changed after overexpression of miR-155-3p (Fig. 7a). Furthermore, gene set enrichment analysis indicated that FBXW7 gene signature was significantly enriched in miR-155-3p overexpression cells (Fig. 7b). These results suggested that miR-155-3p regulates HCC cells proliferation may be mediated by FBXW7.

**Fig. 8:**
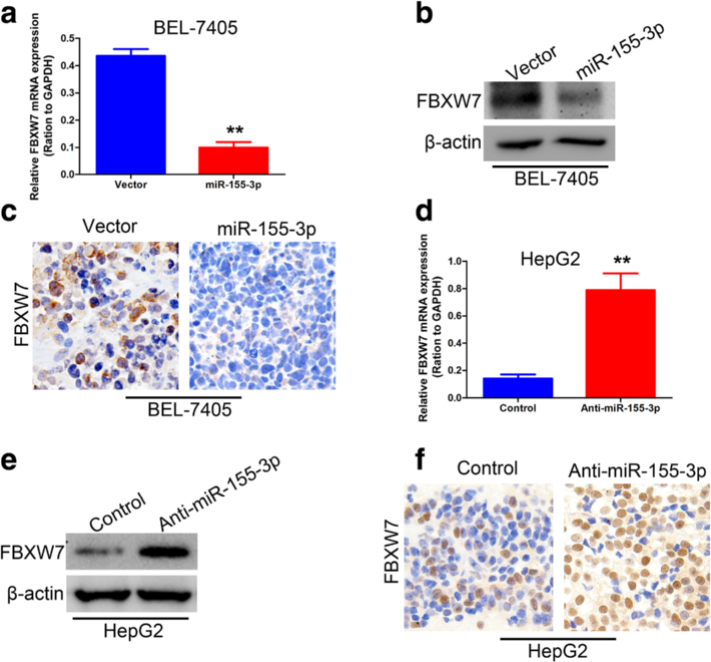
FBXW7 is the potential target of miR-155-3p. **a** The expression of FBXW7 mRNA was analyzed by qRT-PCR in BEL-7405-miR-155-3p and its control cells. **b** Western blot analysis was performed to detect the expression of FBXW7 and internal control β-actin in BEL-7405-miR-155-3p and its control cells. **c** The expression of FBXW7 protein was measured by immunohistochemical analysis in BEL-7405-miR-155-3p and its control cells in vivo tumor samples. Representative results are shown in micrographs. **d** The expression of FBXW7 mRNA was analyzed by qRT-PCR in HepG2-anti-miR-155-3p and its control cells. **e** Western blot analysis was performed to detect the expression of FBXW7 and internal control β-actin in in HepG2-anti-miR-155-3p and its control cells. **f** The expression of FBXW7 protein was measured by immunohistochemical analysis in in HepG2-anti-miR-155-3p and its control cells in vivo tumor samples. Representative results are shown in micrographs. All experiments were performed in triplicate. **P < 0.01, unpaired two-tailed Student’s t-test

To confirm that FBXW7 is a target of miR-155-3p in BEL-7405 and HepG2 cells, the mRNA and protein levels of FBXW7 were analyzed by qRT-PCR and western blot in overexpressed miR-155-3p BEL-7405 cells and inhibited miR-155-3p HepG2 cells. We found that FBXW7 was downregulated in the miR-155-3p overexpressed cells both in mRNA and protein levels (Fig. 8a and b), whereas FBXW7 was increased by the miR-155-3p specific inhibitor compared with that observed in the control cells (Fig. 8d and e). The FBXW7 protein expression was also confirmed by immunohistochemical analysis in vivo tumor tissues (Fig. 8c and f).

**Fig. 9:**
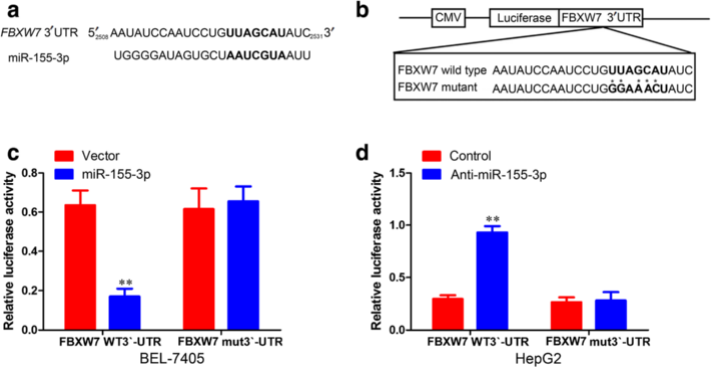
FBXW7 is a direct target of miR-155-3p. **a** The potential binding site for miR-155-3p in 3’UTR of FBXW7 mRNA. **b** FBXW7 3’-UTR possessing a mutation in the putative miR-155-3p binding site. **c** The luciferase activity after transfection in BEL-7405-miR-155-3p and its control cells of the indicated 3’-UTR-driven reporter constructs. Reporter plasmids containing no oligonucleotides as a Control, the wild-type 3’UTR region of FBXW7 as a Wild type and the mutant 3’UTR region as a Mutant. **d** The luciferase activity after transfection in HepG2-anti-miR-155-3p and its control cells of the indicated 3’-UTR-driven reporter constructs. Reporter plasmids containing no oligonucleotides as a Control, the wild-type 3’UTR region of FBXW7 as a Wild type and the mutant 3’UTR region as a Mutant. **P < 0.01, unpaired two-tailed Student’s t-test

We next performed a luciferase reporter assay to assess whether miR-155-3p inhibits the translation of FBXW7. One potential binding site for miR-155-3p was found in the 3’-UTR region of FBXW7 mRNA and FBXW7 3’-UTR possessing a mutation in the putative miR-155-3p binding site (Fig. 9a and b). The detection of a luciferase activity revealed that miR-155-3p significantly suppressed the activity of luciferase combined with wild-type FBXW7 3’-UTR in the BEL-7405 miR-155-3p cells (Fig. 9c) and increase FBXW7 3’-UTR in the HepG2 miR-155-3p cells, whereas no differences were observed following treatment with the control luciferase and FBXW7 3’-UTR possessing a mutation in the putative miR-155-3p binding site (Fig. 9d). These results suggest that miR-155-3p may directly bind to FBXW7 mRNA and regulates the FBXW7 expression via translational inhibition.

### FBXW7 is responsible for clone formation and proliferation ability of BEL-7405 cells and HepG2 cells

**Fig. 10:**
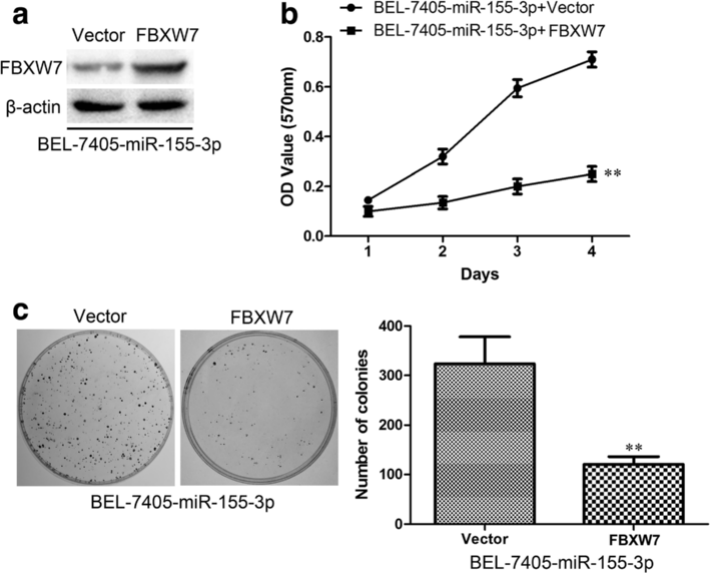
FBXW7 expression suppresses the colony formation and proliferation phenotype of BEL-7405 cells. **a** BEL-7405-miR-155-3p were transfected FBXW7, the results of immunoblotting for FBXW7 and β-actin are shown. **b** BEL-7405-miR-155-3p transfected with FBXW7 show a significant reduction in proliferation in comparison to control cells. **c** BEL-7405-miR-155-3p transfected with FBXW7 reduces colony formation. **P < 0.01, unpaired two-tailed Student’s t-test

In order to investigate whether FBXW7 is responsible for the enhanced clone formation and proliferation ability of BEL-7405 cells, BEL-7405-miR-155-3p cells were transfected with FBXW7 or empty plasmid and FBXW7 expression were analyzed by Western blotting (Fig. 10a). The treated cells were evaluated for tumorigenesis using a MTT assay (Fig. 10b) and colony formation assay (Fig. 10c). Overexpression of FBXW7 strongly inhibited cell proliferation compared to control cells, and decreased colony formation was clearly observed in the cells with FBXW7 overexpression, whereas transfected with empty vector did not affect colony formation.

**Fig. 11:**
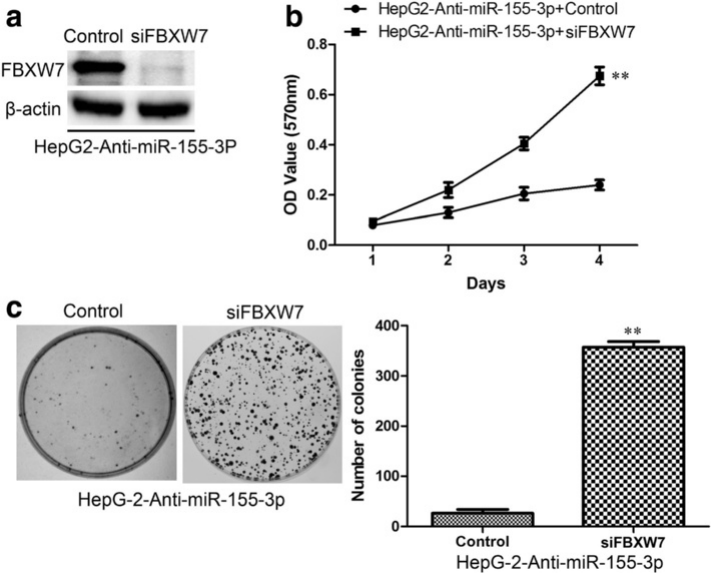
Reduced FBXW7 expression enhances the colony formation and proliferation phenotype of HepG2-Anti-miR-155-3p cells. **a** Knockdown of FBXW7 in HepG2-Anti-miR155-3p cells, the results of immunoblotting for FBXW7 and β-actin are shown. **b** Knockdown of FBXW7 in HepG2-Anti-miR-155-3p cells facilitates the proliferation ability of HepG2-Anti-miR-155-3p cells. **c** Knockdown of FBXW7 in HepG2-Anti-miR-155-3p cells increases colony formation. **P < 0.01, unpaired two-tailed Student’s t-test

The expression of FBXW7 in HepG2-Anti-miR-155-3p cells was suppressed by siRNA and FBXW7 protein levels were analyzed by Western blot (Fig. 11a). The treated cells were evaluated for proliferation property using a MTT assay (Fig. 11b) and colony formation assay (Fig. 11c). Interference of FBXW7 expression strongly enhanced cell proliferation compared to control cells. Increased colony formation was clearly observed in the cells with FBXW7 suppression, whereas treatment with nonspecific siRNA did not affect colony formation. These results suggest that inhibition of FBXW7 expression induced by miR-155-3p is responsible for clone formation and proliferation ability in HCC cells.

## Discussion

Increasing data has indicated miRNAs to be critical regulators in cancer-related processes [30], it’s still largely unknowing the molecular mechanisms by which miRNAs modulate the behavior of cancer cells. Here we demonstrated that upregulation of miR-155-3p was common in HCC tissues and could serve as an independent prognosis predictor for HCC patients. Furthermore, our findings also showed that ectopic expression of miR-155-3p could accelerate clone formation and proliferation ability in HCC cells. In addition, we further identified FBXW7 as a functional target of miR-155-3p and demonstrated an involvement of FBXW7 in the effects of increased miR-155-3p on promoting clone formation and proliferation. Our data suggest that miR-155-3p may play a fundamental role for in clone formation and proliferation of HCC cells, and implicate the potential application of miR-155-3p in cancer prognosis prediction and therapy.

Clone formation and proliferation are major cellular processes that must be circumvented to prevent malignant tumor progression [31]. Upregulation of miR-155-3p may enhance the clone formation and proliferation of a variety of cancer cells, and in turn, stimulate the development of tumors. In this study, we employed BEL-7405 and HepG2 cells to explore how miR-155-3p exerts its function and modulates the clone formation and proliferation of HCC cells. Our findings show that ectopic expression of miR-155-3p in HCC cells could facilitate clone formation and proliferation, and upregulation of miR-155-3p can promote clone formation and proliferation in HCC cells. Furthermore, our data in 45 paired HCC tissues showed that the expression of miR-155-3p was significantly elevated in cancerous tissues compared with juxta cancerous tissues, revealing a clear correlation between miR-155-3p expression and HCC malignancy. Together with the in vitro findings showing that increased miR-155-3p promotes clone formation and proliferation in HCC cell lines, these results further confirmed the roles of miR-155-3p in clone formation and proliferation of HCC cells.

We used the microarray, miRNA target prediction program and a luciferase report assay to demonstrate that miR-155-3p directly down regulates FBXW7 by binding its 3’-UTR. Furthermore, ectopic expression of miR-155-3p resulted in downregulated expression of endogenous FBXW7 protein and mRNA, while targeted knockdown of miR-155-3p increased the expression of FBXW7 protein and mRNA. In addition, we observed significant inverse correlations between miR-155-3p and FBXW7 expression levels in vivo, which supports the regulation of miR-155-3p on FBXW7 observed in vitro. In conclusion, FBXW7 is a direct target of miR-155-3p.

## Conclusions

In the current study, we investigated the potential role of miR-155-3p in HCC tumor progression and its underlying mechanisms. We herein demonstrated that miR-155-3p promotes HCC tumorigenesis via reducing the expression of FBXW7 by inhibiting translation. Our results suggest that upregulation of miR-155-3p may play an essential role in the development of HCC and may be employed as a prognosis marker and therapeutic target of HCC. Nevertheless, these data should be further validated in independent cohorts and prospective trials.

## Abbreviations

DMEM, Dulbecco’s Modified Eagle’s Medium; FBXW7, F-box and WD repeat domain containing 7; HBV/HCV, hepatitis B virus/hepatitis C virus; HCC, hepatocellular carcinoma; MiRNAs, microRNAs; MTT, 3-(4, 5-dimethylthiazol-2-yl)-2, 5-diphenyltetrazolium; NASH, nonalcoholic steatohepatitis; UTR, untranslated region
